# Factors associated with low tuberculosis preventive therapy prescription rates among health care workers in rural South Africa

**DOI:** 10.1080/16549716.2021.1979281

**Published:** 2021-10-15

**Authors:** Amiya A. Ahmed, Megan Grammatico, Anthony P. Moll, Sipho Malinga, Philile Makhunga, Salome Charalambous, Joseph B. Ladines-Lim, Justin Jones, Koeun Choi, Sheela V. Shenoi

**Affiliations:** a Department of Medicine, University of Maryland School of Medicine, Baltimore, MD, USA; b Department of Medicine, University of Connecticut School of Medicine, Farmington, CT, USA; c Church of Scotland Hospital, Tugela Ferry, South Africa; d Philanjalo NGO, Tugela Ferry, South Africa; e Aurum Institute, Johannesburg, South Africa; f Department of Medicine and Pediatrics, Michigan Medicine, Ann Arbor, MI, USA; g Institute of Behavioral Research, Texas Christian University, Fort Worth, TX, USA; h Department of Medicine, Yale University School of Medicine, New Haven, CT, USA

**Keywords:** HIV, implementation, prevention, rural health, tuberculosis

## Abstract

**Background:**

Despite extensive rollout of tuberculosis preventive therapy (TPT) in South Africa to reduce the incidence of tuberculosis among people living with HIV (PWH), rates of initiation and completion have remained suboptimal.

**Objective:**

This study aimed to identify factors associated with low TPT prescription rates among health care workers (HCWs) in rural South Africa.

**Methods:**

A cross-sectional study was conducted using an anonymous 39-item questionnaire guided by the Consolidated Framework for Implementation Research (CFIR). HCWs from a government district hospital and 14 primary healthcare clinics (PHCs) in the rural Msinga sub-district of KwaZulu-Natal were surveyed from November 2019 to January 2020. Self-reported data on prescription rates as well as knowledge, attitudes, beliefs, and practices regarding isoniazid preventative therapy, the current TPT regimen, were obtained. Factor analysis and logistic regression were used to determine associations with low prescription rates (< 50% of PWH) for TPT prescribers, and results were placed within CFIR-driven context.

**Results:**

Among 160 HCWs, the median (IQR) age was 39 (33–46) years, 76% were women, 78% worked at a PHC, and 44% had experience prescribing TPT. On multivariable analysis, prescribers (n = 71) who believed their patients would not disclose TPT use to others were significantly less likely to prescribe TPT (aOR 4.19 95% CI 1.35–13.00; p = 0.01). Inadequate isoniazid supplies trended towards significance (aOR 10.10 95% CI 0.95–106.92; p = 0.06) in association with low prescription rates.

**Conclusions:**

Strengthening HCW training to emphasize TPT prescription to all eligible PWH regardless of beliefs about patient disclosure and ensuring a consistent isoniazid supply at the health systems-level are both critical steps to enhancing TPT implementation in rural South Africa.

## Background

In 2011, the World Health Organization (WHO) released guidelines endorsing the use of tuberculosis preventive therapy (TPT) for preventing tuberculosis in resource-limited settings with high burdens of tuberculosis (TB) and human immunodeficiency virus (HIV) infection [1]. The government of South Africa quickly adopted these guidelines in its own policy to increase uptake of TPT and ease the country’s burden of TB and HIV [2], which is among the highest in the world [3,4]. However, while South Africa has led global implementation, rates of both initiation and completion of TPT have remained low [5,6]. In 2019, nearly a decade after TPT implementation in South Africa, only 69% of newly enrolled HIV-positive patients in South Africa were prescribed TB preventive treatment (TPT) [3,7], which falls short of the expectations of the current South African guidelines that state all new eligible people living with HIV (PWH) are expected to receive TPT immediately alongside antiretroviral therapy (ART) initiation [2].

Several studies have investigated the factors associated with TPT implementation among patients and community members [8,9]. Among PWH in rural KwaZulu-Natal, South Africa, self-reported barriers include poor understanding of TPT’s preventative role, inefficiencies during healthcare delivery, such as long queues, and the expenses associated with access to healthcare, such as the cost of transportation and opportunity cost of lost wages. Conversely, adequate knowledge of TB and TPT, acceptance of one’s HIV diagnosis, social support in the community, and the ability to communicate comfortably with a health care worker (HCW) were all identified as facilitators [8]. Elsewhere in Kwazulu-Natal Province, South Africa, TPT was perceived by community members to be dangerous because of the unsustainable costs, stigma of pill consumption, and the belief that it introduced excess dirt or toxins, locally referred to as *ukungcola*, into the body [9].

Few studies have investigated the facilitators and barriers to TPT implementation from the perspective of HCWs. In Gauteng Province, South Africa, a qualitative study found that HCWs reported the primary barrier to TPT implementation was HCW knowledge regarding TPT. While all doctors in the study had heard of TPT, there were many who were either unfamiliar with or in disbelief of its efficacy in preventing TB [10], resulting in limited TPT use. In a more recent South African study, nurses were placed in a clinical mentorship program to improve TPT prescription rates. Participants from this study reported high adoption and acceptability of TPT, but identified that national TPT guidelines were a significant barrier to TPT initiation due to their ambiguous nature [11]. A quantitative multi-site, cross-sectional study conducted in Ethiopia found that a clinician’s perception that active TB was difficult to rule out was independently and inversely associated with high TPT rates [12].

The Consolidated Framework for Implementation Research (CFIR) is an established implementation science framework for assessing health interventions within the appropriate context. Contextual factors may influence either delivery, outcomes, or how the interventions perform and are critical to understanding how to strengthen implementation. CFIR’s core constructs provide a roadmap for evaluating all aspects of implementation within five major domains, including 1) the ‘intervention’, 2) ‘individuals’, 3) ‘inner setting’, 4) ‘outer setting’, and 5) the ‘processes’ of delivery [13]. All of the barriers mentioned in the previous paragraphs would fall into one of these five domains. For example, gaps in knowledge regarding TPT may fall within the ‘individual’ domain, while the distance from a patient’s home to the clinic could fall within the ‘outer setting’ domain, By recognizing the context from which these facilitators and barriers emerge, a broader framework can be established to improve implementation.

Therefore, in the context of suboptimal TPT implementation in South Africa, previous evaluations of patients’ perspectives, and the limited number of studies investigating HCW-related associations with low TPT rates, we sought to use CFIR to examine the factors associated with low TPT prescription rates among HCWs in the rural Msinga municipality of KwaZulu-Natal, South Africa.

## Methods

### Study setting

The rural Msinga municipality in KwaZulu-Natal, South Africa has a total area of ~2500 square kilometers and is home to a population of nearly 180,000 traditional Zulu people [14]. The municipality is among the poorest in the nation with high rates of unemployment (85%) and nearly 50% of the population lacking access to electricity [15]. The region is characterized by a high TB incidence (> 1100/100,000) and HIV antenatal prevalence (31.1%) [6], and 94.2% of TB/HIV co-infected clients are on ART [16]. A government district hospital and 16 satellite primary health care clinics (PHCs), run by senior level professional nurses who have prescribing capacity, provide free primary care services, including ART and TPT to HIV-positive patients in the sub-district [8,15,16]. While TPT may refer to various TB preventive regimens, the only option for TPT at the time of this study was 6–12 months of daily isoniazid, which has been implemented since May 2010 [2].

### Study participants and study period

Health-care workers (HCWs), ≥18 years of age who worked in a public-sector health facility in the Msinga sub-district with direct patient care responsibilities, were included in the study. Potential participants were recruited at clinic staff meetings November 2019 to January 2020. All clinic site visits were scheduled in advance with and approved by the operational manager of each individual clinic.

### Definitions

#### Direct *TPT providers*

*Direct* TPT providers were defined as medical officers, professional nurses, and staff nurses who self-identified by providing a positive response to the survey question: ‘Do you have experience prescribing TPT?’ These HCWs were involved in identifying eligible patients and/or prescribing TPT. Medical officers have a MBBCH, MBBS, or MD/DO degree and are registered with the Health Professions Council of South Africa, professional nurses have a baccalaureate degree in nursing and are registered with the South African Nursing Council, and staff nurses have a college diploma in nursing. Of note, staff nurses are not allowed to sign a prescription, but are heavily involved in the multistep process of TPT initiation.

#### Indirect *TPT providers*

*Indirect* TPT providers were defined as all other participants in our study who did not fit the definition of a *direct* TPT provider. The participants included professional nurses, staff nurses, voluntary counseling and testing (VCT) counselors, and other clinicians who provided a negative response to the above question. Of note, the few participants who self-identified as a prescriber, but did not identify as a medical officer, professional nurse, or staff nurse were defined as an *indirect* TPT provider. The participants may screen, educate, and counsel patients about preventive therapy.

### Data collection

An anonymous, 39-question survey tool was used to collect self-reported data. There were six questions about demographics, and 33 questions about knowledge, and perceptions regarding HIV and TB, as well as pre-existing knowledge, attitudes, practices, and beliefs towards isoniazid preventive therapy, the current TPT regimen. The survey included ‘Yes/No/Unsure’ questions as well as Likert scale items with the following possible answer choices: (1) ‘A great deal/A lot/Some/A little/Nothing’ or (2) ‘Every patient/More than half of my patients/Less than half of my patients/Never/Declined to Answer’. The survey tool explored all five CFIR domains [13]. The survey tool was made available in English and isiZulu. A copy of the survey tool has been included in **Appendix 1**.

### Outcome

The outcome in this study was a low TPT prescription rate, which was defined as prescribing to < 50% of eligible PWH. Our outcome was defined retrospectively using Question 12 in the survey tool (**Supplemental Information Item 1**).

### Data analysis

Exploratory factor analysis (EFA) was performed six times separately on the six domains of the survey in order to reduce the number survey items for more concise analysis and discussion. Baseline characteristics and EFA-generated bivariate scores were compared between indirect and direct TPT providers using Pearson’s Chi-square analysis and Mood’s median test, as appropriate. Unadjusted binary logistic regression was used to calculate odds ratios for each factor independently to estimate associations with the outcome of interest. A multivariable binary logistic regression model adjusting for potential predictors was then constructed, including all statistically significant co-variates from the univariate analysis. Statistical tests were two-tailed with 95% confidence intervals. P-values < 0.10 were considered significant for univariate analysis to ensure a broad range of factors were being considered. A stricter p-value cut-off of < 0.05 was considered significant for final multivariate analyses. SPSS 25 for Windows (2017; Armonk, NY: IBM Corp.) was used for all data analyses.

### Defining scales and scores

For each factor extracted, a 2 to 4-item scale was generated, based on the number of questions that contributed to the factor. For example, a 4-item ‘perceived patient stigma towards IPT’ scale (values 0–4) was initially created. Next, from each scale, a bivariate score was created. Therefore, from our above scale, a ‘perception of increased patient stigma towards IPT’ score was defined as having a value of > 0. This was performed for all 13 factors. A list with the exact question contributions to each individual factor is included in the **Supplemental Information Item 1.**

### Ethics approval

The study protocol was approved by the Yale University’s Human Investigation Committee and the University of KwaZulu-Natal Biomedical Research Ethics Committee.

## Results

### Descriptive characteristics

There were 160 HCWs in our study with a median (± IQR) age of 39 (±13) years, 76% were women, and 44% were direct TPT providers (Table 1). The majority (78%) were from a primary health care clinic as opposed to the district hospital. Direct TPT providers were more likely to be older and either a professional nurse or a medical officer.

**Table 1.** Descriptive characteristics of indirect and direct TPT providers

Variables N (%) or median (±IQR)	All respondents (n = 160)	Indirect TPT providers (n = 83)	Direct TPT providers (n = 71)	P-value
Age, years	39 (± 13)	38 (± 11)	41.5 (± 16.75)	**0.01**
Female	75.6% (121/159)	73.5% (61/83)	78.6% (55/70)	0.46
Primary healthcare clinics	78.1% (125/160)	79.5% (66/83)	74.6% (53/71)	0.47
District hospital	21.9% (35/160)	20.5% (17/83)	25.4% (18/71)	0.49
Professional nurse	37.5% (60/160)	16.9% (14/83)	62.0% (44/71)	**< 0.01**
Staff nurse	31.9% (51/160)	41.0% (34/83)	23.9% (17/71)	**0.03**
VCT counselor	10.6% (17/160)	19.3% (16/83)	0% (0/71)	**< 0.01**
Medical officer	6.3% (10/160)	0% (0/83)	14.1% (10/71)	**< 0.01**
Other position	13.1% (21/160)	22.9% (19/83)	0% (0/71)	**< 0.01**

### Exploratory factor analysis

**Figure 1.**
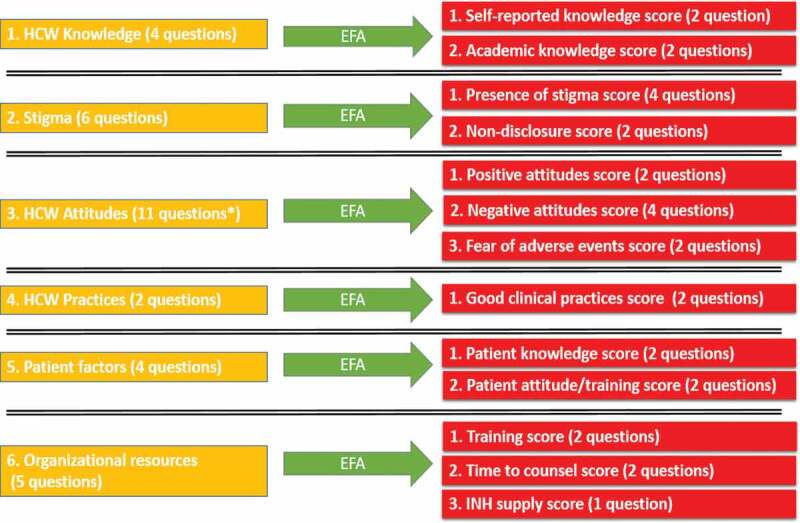
Flowchart of factors generated by exploratory factor analysis (EFA) of survey questions

Factor analysis was performed separately on the six domains from the survey. Variable reduction was carried out using principal axis factoring and orthogonal varimax rotation with Kaiser Normalization. KMO’s and Bartlett’s test were used to determine if EFA was appropriate. Factors extracted had eigenvalues > 1 and communalities > 0.50. Aside from one item used as the outcome, 32 survey items were reduced to a total of 13 factors as seen in Figure 1.

### Knowledge, attitudes, and beliefs of direct TPT providers

Regarding knowledge, the majority (90%) of direct TPT providers (n = 71) self-reported knowing ‘a lot’ or ‘a great deal’ about TPT (Table 2). However, a smaller majority (54%) answered objective questions correctly when tested on current South African TPT guidelines. In terms of attitudes, nearly all (99%) had a positive attitude concerning the effectiveness of TPT. However, that did not rule out having concerns as well as some negative attitudes towards TPT among study participants.

**Table 2.** Knowledge, attitudes, and beliefs among direct TPT providers (n = 71)

Themes	Factors	Frequency, % (n/N)
I. HCW Knowledge of TPT	Good self-reported TPT knowledge	90.0% (63/70)
	Good knowledge of TPT guidelines	53.7% (36/67)
II. HCW Beliefs on Patient Stigma concerning TPT	Perception of increased patient stigma towards TPT	60.0% (42/70)
	Perception of poor patient comfort with TPT disclosure	60.6% (43/71)
III. HCW Attitudes concerning TPT	Concern about TPT adverse events	77.5% (55/71)
	Positive attitude towards TPT	98.6% (68/69)
	Negative attitude towards TPT	62.9% (44/70)
IV. HCW Beliefs on Patient Factors concerning TPT	Good patient knowledge of TPT	88.6% (62/70)
	Good patient attitude & practices towards TPT	90.1% (64/71)
V. Organizational Level Factors concerning TPT	Sufficient training to counsel patients on TPT	81.7% (58/71)
	Sufficient time to counsel on TPT	95.8% (68/71)
	Sufficient isoniazid supply	93.0% (66/71)

### Univariate associations with low TPT prescription rates

Among direct TPT prescribers (n = 71), none of the descriptive characteristics, including age, sex, length of time practicing as a HCW, or years managing PWH, were associated with low prescription rates. While poor objective knowledge of TPT-related guidelines had no association, poor self-reported knowledge did trend towards significance (p = 0.06). A perception among health workers of poor patient comfort with TPT disclosure (p = 0.01), insufficient TPT training (p = 0.04), and inadequate isoniazid supplies (p = 0.04) were significantly associated with lower prescription rates (Table 3). On the other hand, HCWs’ attitudes concerning TPT and patient factors concerning TPT had no association with lower TPT prescription rates.

**Table 3.** Factors associated with low TPT prescription rates (n = 71)

	Odds ratios (95% confidence intervals)	
	Unadjusted OR (95%CI)	Multivariable adjusted OR (95%CI)*
Demographics		
Age, years	0.99 (0.94–1.04) p = 0.68	**
Female	0.55 (0.16–1.83) p = 0.33	**
Length of time as a healthcare worker, years	0.99 (0.94–1.04) p = 0.67	**
Length of time managing HIV, years	1.01 (0.93–1.10) p = 0.78	**
HCW Knowledge of TPT		
Poor self-reported TPT knowledge	5.41 (0.91–32.29) *p = 0.06*	**
Poor knowledge of TPT guidelines	0.61 (0.22–1.70) p = 0.34	**
HCW Beliefs on Patient Stigma concerning TPT		
Perception of increased patient stigma towards TPT	1.43 (0.49–4.18) p = 0.52	**
Perception of poor patient comfort with TPT disclosure	4.71 (1.61–13.38) *p = 0.01*	4.19 (1.35–13.00) *p = 0.01*
HCW Attitudes concerning TPT		
Concern about TPT adverse events	0.62 (0.19–2.01) p = 0.42	**
Negative attitude towards TPT	2.90 (0.74–11.41) p = 0.13	**
Patient Factors concerning TPT		
Perception of poor patient TPT knowledge	3.26 (0.66–16.05) p = 0.15	**
Perception of poor patient attitude & practices towards TPT	1.74 (0.35–8.52) p = 0.50	**
Organizational Level Factors concerning TPT		
Insufficient training	4.01 (1.11–14.57) *p = 0.04*	**
Insufficient time to counsel	4.70 (0.40–54.84) p = 0.22	**
Insufficient isoniazid supply	10.44 (1.09–99.86) *p = 0.04*	10.10 (0.95–106.92) P = 0.06

*Adjusted for poor self-reported knowledge, perception of poor patient comfort with TPT disclosure, insufficient training, and insufficient isoniazid supply

### Multivariate associations with low TPT prescription rates

After multivariable adjustment, only one factor was significantly associated with low prescription rates (Table 3). Direct TPT providers who perceived their patients to have poor disclosure of TPT use were approximately four times more likely to have a low prescription rate, defined as prescribing TPT to < 50% of their patients. Of note, inadequate supplies of isoniazid trended towards significance in the multivariate model (aOR 10.10 95% CI 0.95–106.92; p = 0.06).

### Findings within the context of the CFIR model

**Figure 2.**
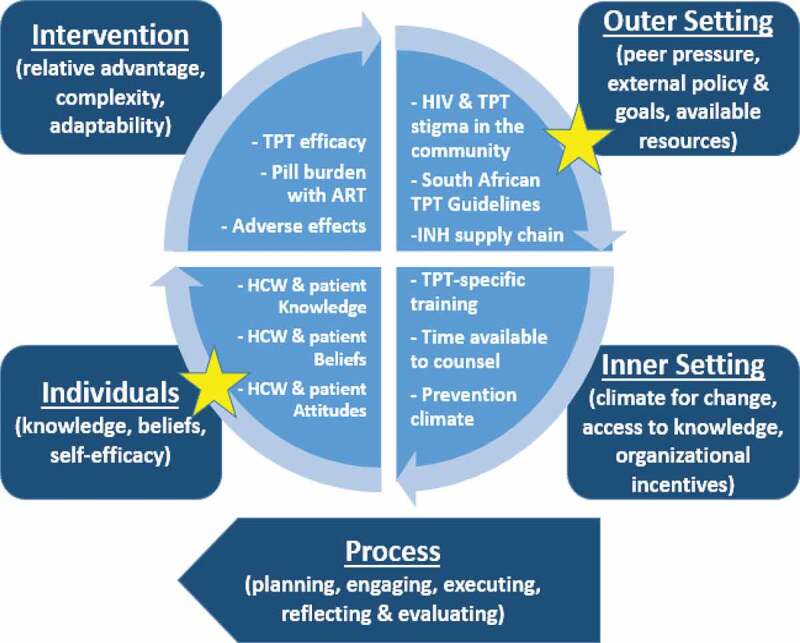
CFIR model of TPT implementation in rural South Africa. Domains most likely to benefit from further refinement are marked with yellow stars (adapted from *Damschroder et al*.)

Within the CFIR framework, our results highlight two of the five major domains: the outer setting and the characteristics of individuals involved (Figure 2). The perception of HCWs regarding patient comfort with TPT-use disclosure is a modifiable factor at the individual level. On the other hand, the inadequate resources in regards to TPT, including insufficient isoniazid, demonstrates room for improvement at the level of the outer setting in regards to TPT implementation.

## Discussion

To the best of our knowledge, this is the first study to quantitatively examine HCW-related factors associated with low TPT prescription rates in rural South Africa. We identified that perception of patient willingness to disclose use of TPT influenced HCWs’ likelihood of prescribing TPT to the majority of their patients. Furthermore, the data suggested that HCWs’ belief that isoniazid is not always readily available also affected TPT prescription rates in our study. These findings are important as they provide necessary insight regarding potential areas of improvement at the provider and organizational levels for successful TPT implementation in a rural setting.

In our study, we found that prescribers who believed their patients were unlikely to disclose their TPT use had significantly lower prescription rates of TPT. The effect of disclosure on adherence to HIV care has been well documented in the literature [17,18]. Disclosure of HIV status has been shown to contribute to ART adherence through improving social support, financial support, and reductions in anxiety and depression, among other mechanisms [17]. HIV-related stigma has been strongly linked to fear of TPT disclosure and TPT-related stigma [8,13,19–21]. To that end, studies in Eswatini and South Africa have found disclosure of HIV status to also improve TPT adherence through mechanisms similar to those stated above [19,20]. While there are fewer such studies focusing on disclosure of TPT use in particular, Adams *et al*. determined that disclosure of TPT improved TPT completion in Eswatini [17]. Participants in their 2019 study reported that TPT disclosure supported adherence through medication reminders from family members and being able to openly take their medications [17]. To our knowledge, there are no studies documenting the association between HCW perception of patient disclosure and TPT prescription rates. However, previous studies from Ethiopia and South Africa have shown that the fear of generating isoniazid resistance secondary to poor patient adherence were primary reasons HCWs were reluctant to offer TPT [11,22]. While it is possible that fear of TB drug resistance secondary to the suspicion of poor patient disclosure contributes to low prescription rates in this study, only a quarter of prescribers in our study expressed concerns for TB drug resistance due to TPT use. Further investigations are necessary to clarify the reasons behind concerns about disclosure including the potential role of stigma among health care workers.

Additionally, while isoniazid supply shortages were only trending towards significance in association with low prescription rates, nearly a quarter of our participants reported not having TPT medications available when needed. This is in line with the current literature, as limited isoniazid supplies are well documented as a barrier to TPT implementation [22–24]. A 2020 multi-site study in Nigeria identified stockouts of isoniazid as a reason for low TPT uptake and suggested the need for strengthening drug supply logistics in order to optimize TPT uptake [23]. Isoniazid stockouts serve as a two-fold barrier. First, the obvious absence of medications prevents HCWs from supplying their patients with TPT. Second, a lack of certainty in having an uninterrupted supply of isoniazid has an added effect of generating HCW fear of creating isoniazid resistance [22]. Though fear of isoniazid resistance was incorporated into our ‘negative attitude towards TPT’ score, which had no association with low TPT prescription rates, it is still worth noting 25.4% (18/71) direct TPT providers had a fear of TB resistance as a result of TPT. Therefore, appropriate capacity building to address inconsistencies in the supply of isoniazid may be needed to improve confidence among HCWs and increased prescription.

Context is a key component of evaluation implementation, and placing these two factors within a theoretical framework, such as the CFIR model shown in Figure 2, enables us to bridge data to motivating behavioral change through potential health policy implementation [20]. As mentioned, our study identifies two specific levels for change. At the individual level, while HCWs should endorse patients’ disclosure of their health status to facilitate social support, it is critical to support HCWs in prescribing TPT to all eligible PWH regardless of beliefs about patient disclosure as TPT is a lifesaving tool [21]. At the level of the outer setting, policymakers should ensure a consistent supply of isoniazid. In this rural part of South Africa, these were the two most pertinent domains to improving implementation, and changes at both levels are necessary to successfully strengthen TB prevention health systems. Though not significantly associated with TPT prescription rates on adjusted analyses, it is prudent for policymakers to also ensure robust HCW training and public health education about TPT for patients and community members given its demonstrated mortality benefit independent of ART.

It is worth noting factors that have been found to be significant in previous studies, and which were also included in our study but not found to be associated with poor TPT prescription rates. Both a lack of TPT knowledge and training have been found to be associated on unadjusted analysis with poor practices regarding TPT provision among clinicians in urban South Africa and Nigeria [25–27]. Similarly in our rural study, poor HCW knowledge and training were associated with low prescription rates on univariate analysis, but not identified as barriers after adjusted analysis. Furthermore, evidence has demonstrated that direct providers’ attitudes influence their practices concerning TPT [11,25]. While we found that providers’ attitudes did not impact prescription rates, our study did align with the literature demonstrating that HCWs generally found TPT effective and endorsed TB prevention [17,25,27].

Several limitations of this study must be acknowledged. First, selection bias may have been present as clinics were visited at select times as determined by the operational managers to avoid unnecessary interference with clinical duties. Information bias may have also been present due to missing data as not all participants completed the survey in its entirety. Next, while these findings should be confirmed in a larger sample of HCWs, these findings provide important insight into factors influencing TPT implementation and warrant further evaluation of this critical context. Another limitation was that TPT prescribers as a group were not defined in the traditional sense, and also included staff nurses as they played a key role in TPT initiation. The inclusion of staff nurses in the prescriber group had the potential to attenuate differences observed between prescribers and non-prescriber groups. However, this was not observed, which in fact further strengthens these findings. Additionally, these findings are relevant to primary care clinics in rural resource-limited settings and may not be generalizable to urban areas or other types of health care facilities. Lastly, it is possible that these findings may not be applicable to use of emerging shorter course TPT regimens.

## Conclusion

Supporting HCWs at the individual level to expand TPT prescription, regardless of beliefs about patient disclosure, is critical to implementation. Addressing inconsistencies in the isoniazid supply chain at the health systems-level is also necessary to influence successful implementation of TPT.

## Supplementary Material

Supplemental Material

## Paper context

TB preventive therapy has been shown to reduce mortality among people living with HIV, independent of antiretroviral therapy. However, global implementation lags. Using a validated implementation science framework, this study demonstrates health care workers who believe their patients do not disclose use of TB preventive therapy were significantly less likely to prescribe it, which is modifiable. Therefore, strengthening health care worker training to facilitate prescribing preventive therapy to all eligible patients is critical to implementation efforts.
