# Supplementary figures and images for: Factors associated with low tuberculosis preventive therapy prescription rates among health care workers in rural South Africa
Source: Glob Health Action. 2021 Oct 15;14(1):1979281. doi: 10.1080/16549716.2021.1979281 (PMC8525921; doi:10.1080/16549716.2021.1979281)

# **Supplemental online material**

**Item 1.** 62-item survey tool for data collection


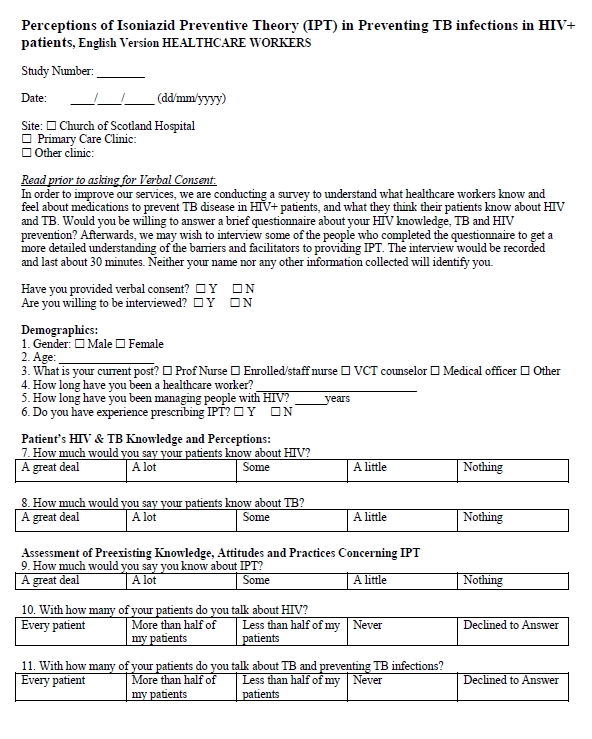


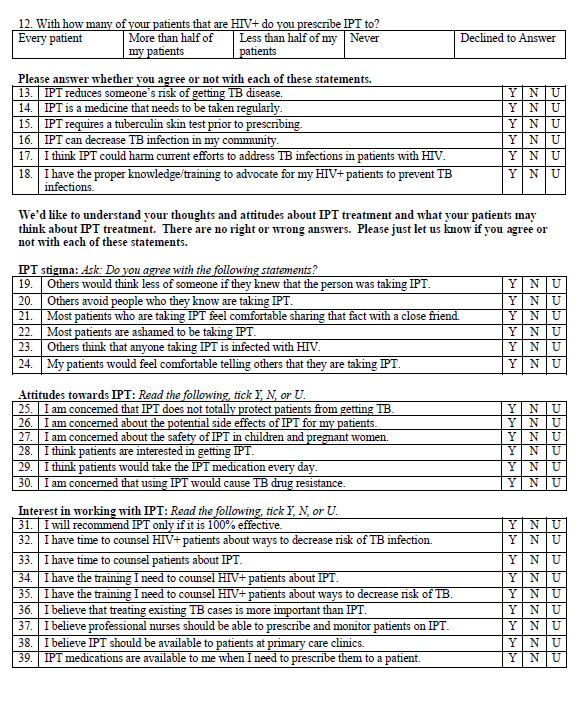


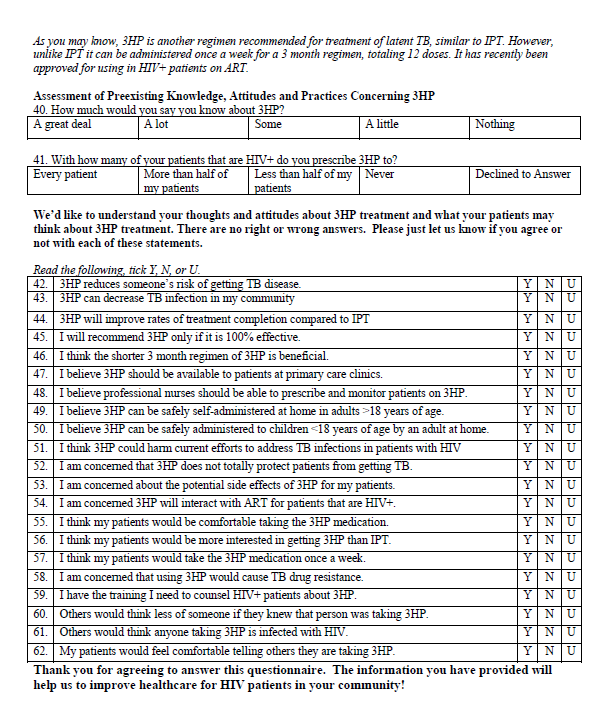

Supplement: Supplemental Material [file ZGHA_A_1979281_SM3338.docx]
